# Bile salt hydrolases: Gatekeepers of bile acid metabolism and host-microbiome crosstalk in the gastrointestinal tract

**DOI:** 10.1371/journal.ppat.1007581

**Published:** 2019-03-07

**Authors:** Matthew H. Foley, Sarah O’Flaherty, Rodolphe Barrangou, Casey M. Theriot

**Affiliations:** 1 Department of Population Health and Pathobiology, College of Veterinary Medicine, North Carolina State University, Raleigh, North Carolina, United States of America; 2 Department of Food, Bioprocessing, & Nutrition Sciences, North Carolina State University, Raleigh, North Carolina, United States of America; University of Wisconsin Medical School, UNITED STATES

## Introduction

**Fig 1:**
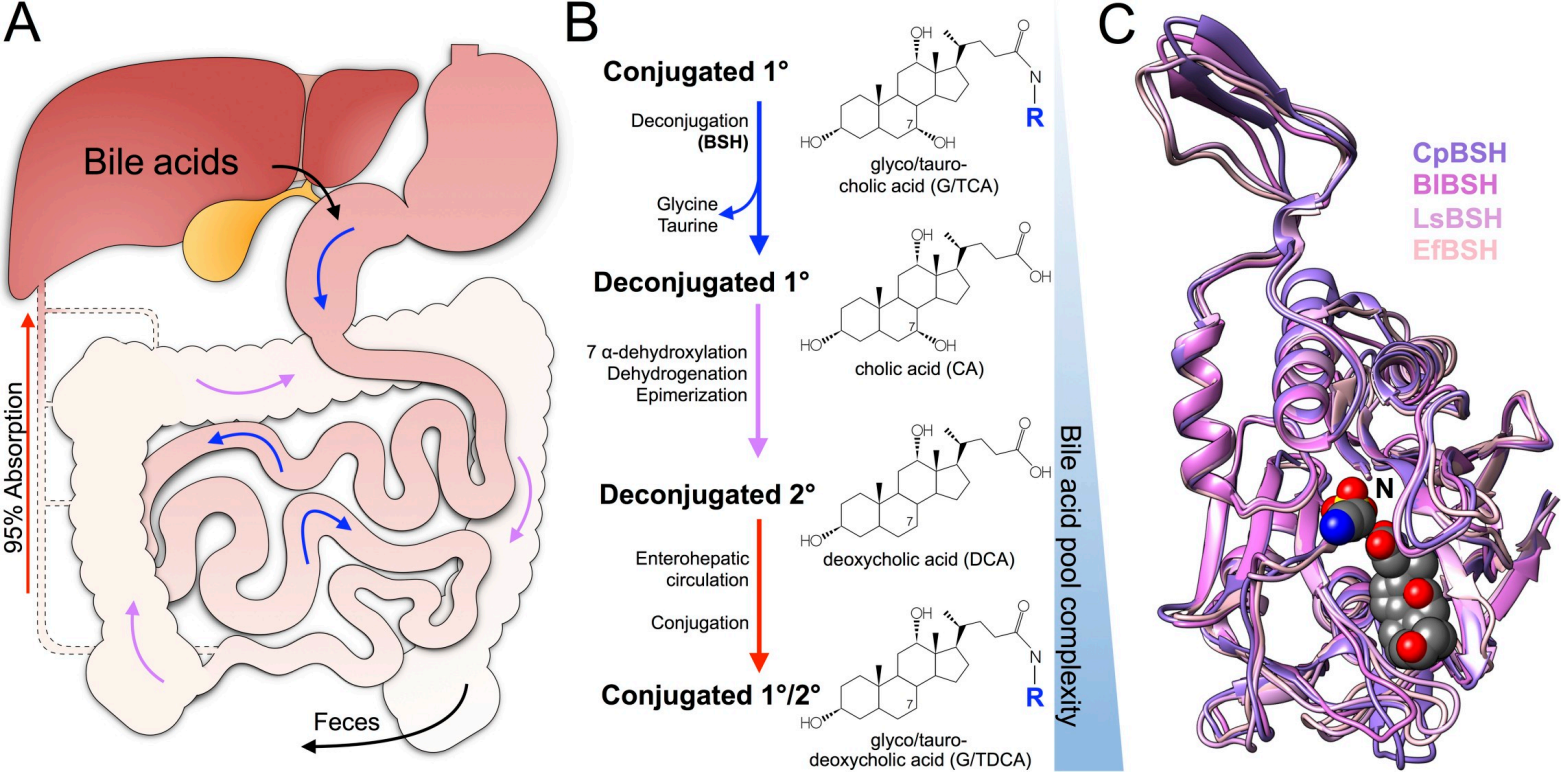
Bile salt hydrolases act on circulating conjugated bile acids in the gut-liver axis. (A) Bile acids synthesized in the liver and stored in the gall bladder enter the small intestine through the duodenum where they reach millimolar concentrations. The majority of bile acids (95%) are reabsorbed in the ileum and recirculate to the liver through the portal vein. The remaining population transit to the colon as they continue to be reabsorbed, and a small (<5%) amount exit through the feces. Recirculating bile acids access host tissues outside the intestines to impart systemic effects on host physiology. (B) BSHs cleave the amide bond in conjugated bile acids to open up the bile acid pool to increased complexity. The gut microbiota performs additional chemistry on deconjugated bile acids to generate the secondary bile acid pool, which can undergo enterohepatic circulation and be reconjugated in the liver. These transformations are illustrated to the right as conjugated CA is deconjugated, subjected to 7 α-dehydroxylation to become DCA, and subsequently reconjugated. (C) Monomeric BSH overlay from *Bifidobacterium longum* (PDB ID 2HEZ), *Enteroccocus faecalis* (PDB ID 4WL3), *Lactobacillus salivarius* (PDB ID 5HKE), and *Clostridium perfringens* (PDB ID 2BJF). Hydrolyzed TDCA in the CpBSH active site is coordinated by several loops that contain the most variation in the peptide backbone compared to the other structures. BSH, bile salt hydrolase; CA, cholic acid; CpBSH, *C*. *perfringens* BSH; DCA,; TDCA, taurodeoxycholic acid; PDB ID, Protein Data Bank ID.

Research on bile acids has increased dramatically due to recent studies demonstrating their ability to significantly impact the host, microbiome, and various disease states [1–3]. Although these liver-synthesized molecules assist in the absorption and digestion of dietary fat in the intestine, their reabsorption and recirculation also gives them access to peripheral organs [4] (Fig 1A). Bile acids serve as substrates for bile acid receptors (BARs) found throughout the body that control critical regulatory and metabolic processes and therefore represent an important class of bioactive molecules [5]. Despite the importance of bile acids to host health, there remain gaps in our knowledge about the bacterial enzymes driving their composition and modification.

Members of the gastrointestinal tract (GIT) microbiota initiate bile acid metabolism via a critical first step catalyzed by bile salt hydrolases (BSHs) [6]. These enzymes hydrolyze and deconjugate the glycine or taurine from the sterol core of the primary bile acids, cholic acid (CA), and chenodeoxycholic acid (CDCA) (Fig 1B). Deconjugated bile acids can subsequently undergo a variety of microbiota-encoded transformations (i.e., 7 α-dehydroxylation, dehydrogenation, and epimerization) that generate secondary bile acids, which have widespread effects on the host and resident microbiota [5, 6]. As the sole enzymes responsible for the pivotal deconjugation reaction, BSH activity serves as a gatekeeper to subsequent bile acid transformations [7]. Therefore, BSH enzymes are a promising tool for targeted manipulation of the microbiota [8]. In this Pearl, we explore what is currently known about BSH enzymes and discuss the recent work showing how their activity has the potential to impact the microbiome, host physiology, and disease outcomes in the GIT.

### The structure and function of BSHs

A recent review by Dong and colleagues has reported in depth on many of the biochemical and structural features of BSHs that are summarized here [9]. BSHs belong to the Ntn (N-terminal nucleophile) superfamily of enzymes, which depends on an N-terminal processing event to reveal the principal catalytic cysteine. This cysteine is buried within the active site that is formed within the conserved αββα core of all Ntn enzymes. Five additional catalytically important residues are strictly conserved across all BSHs (Arg18, Asp21, Asn82, Asn175, Arg228) [10], and it is thought some may assist in the formation of a tetrahedral intermediate between the cysteinyl sulfur and the bile acid amide bond by stabilizing an oxyanion hole—a known catalytic mechanism of other Ntn enzymes [11].

Despite the conservation of their active sites and the similarity of their overall topology (Fig 1C), BSHs have widely different catalytic efficiencies and substrate preferences. BSHs are predominantly expressed in the bacterial cytoplasm as homotetrameric proteins, but examples of extracellular and other oligomeric forms have been observed [3]. The pH optima of most BSHs fall into an acidic range of approximately pH 4.5–6.0. This may reflect BSH acclimatization to the more acidic environment of the proximal GIT, in which conjugated bile acids (that are relatively weak acids), and BSH-encoding bacteria are more abundant. BSH preferences are generally skewed to favor either glyco- or tauro- conjugates, whereas the identity of the sterol core is weighed less heavily [12, 13]. Of the four solved BSH crystal structures [10, 14–16], only the *Clostridium perfringens* BSH (Protein Data Bank ID 2BJF) has been captured with a hydrolyzed taurodeoxycholic acid (TDCA) substrate in its active site [10]. Even with the numerous efforts to characterize different BSHs, the lack of detailed structure-function studies has limited our understanding of the BSH-bile acid interaction and restricted our ability to predict or improve substrate specificity.

### How do BSHs shape the GIT microbiota?

The broad distribution and abundance of BSHs in the GIT suggests that bile acid deconjugation is a selected adaptive trait of several bacterial species for symbiosis or pathogenesis within the host [17, 18]. There has been great interest in surveying BSH activity from strains of gram-positive *Lactobacillus* and *Bifidobacterium* because these species are associated with deconjugation in the small intestine and harboring a BSH is thought to be a beneficial feature of probiotics [7, 18, 19]. Other gram-positive genera such as *Clostridium* and *Enterococcus* similarly display a high occurrence of BSH-positive strains. Recent work characterizing BSH encoding members of the Bacteroidetes phylum has begun to elucidate the phenotypes of gram-negative BSH activity [20, 21].

Despite their single hydrolytic function, BSHs can be advantageous in various ways. Because some bile acids are toxic molecules due to their acidic nature and detergent-like properties [22, 23], BSH activity has been attributed to the detoxification of these damaging effects [24, 25]. The possibility that released amino acids are a source of nutrients [26] or that BSHs promote survival through modification of the bacterial membrane architecture [27] has been proposed as well.

Bacterial pathogens, like some commensals, use BSHs to persist and survive during host infection. *Brucella abortus* BSH activity leads to membrane modifications resisting phagocytosis [28]. *Listeria monocytogenes* exploits its BSH for survival in the bile acid-rich liver and gall bladder [25, 29]. Alternatively, BSH activity can provide protection from pathogens. Microbiome-encoded BSHs and secondary bile acid metabolism are associated with colonization resistance against pathogens such as *Clostridioides difficile*, which can use taurocholate (TCA) as a spore germinant, but vegetative cells are sensitive to most secondary bile acids [30]. Similarly, *Lactobacillus johnsonii* BSH activity has been shown to limit the eukaryotic pathogen *Giardia duodenalis* in vivo [31]. The variety of functions that BSH activity provides is unique to the biology of each organism and the niche it inhabits throughout the GIT, therefore warranting a better understanding of their utility for shaping the microbiota.

The bile acid pool, influenced in part by microbial-encoded BSHs, in turn shapes the microbiome composition and function [32]. This bidirectional relationship can occur directly through inhibiting the growth of bile acid susceptible species or by inducing the germination of others. Indirectly, bile acids can modulate the expression of host intestinal innate immune factors such as intestinal antimicrobial peptides and inducible nitric oxide synthase (iNOS) that select for certain gut inhabitants [33, 34]. Deconjugated bile acids alone have been shown to drive phylum level shifts favoring the firmicutes at the expense of the bacteroidetes [35]. Indeed, bile acid-induced community changes may be an overlooked factor of human diseases that are rooted in alterations of the gut microbiome [12].

### How do BSHs alter host physiology in health and disease?

During enterohepatic recirculation, circulating bile acids interact with BARs in all major organs to impart signals that regulate metabolic and homeostatic processes inside and outside of the gut, in which their transformations originate [12]. The nuclear receptor (NR) farnesoid X receptor (FXR) is the best studied due to its prominence in the gut and liver, and its role in regulating host bile acid synthesis and glucose, lipid, and cholesterol metabolism. Other NRs, as well as G-protein-coupled receptors (i.e., TGR5) also participate in important bile acid signaling. These receptors display a range of affinities for each bile acid within the pool, which can either act as agonists or antagonists for receptor signaling. In this way, BARs can distill the information contained within the entire bile acid pool into a single message.

Several studies have used mouse models to bridge the gaps between weight gain and/or obesity, the microbiota, and bile acids through the FXR signaling. Mouse models of diet-induced obesity have demonstrated that both the microbiome and FXR signaling are necessary for weight gain, implicating deconjugated primary and secondary bile acids as regulators of host metabolism [19, 36]. Furthermore, *Escherichia coli* engineered to express *Lactobacillus* BSHs were able to mimic these effects by modifying the bile acid pool, curbing lipid and cholesterol metabolism, and protecting mice from weight gain [34]. Similar physiological effects were seen using a *Bacteroides thetaiotaomicron* native BSH to modify the germfree mouse bile acid pool in a manner that reflects the BSH’s in vitro substrate preferences, further supporting the notion that these enzymes can be delivered in a targeted manner to selectively manipulate the bile acid pool [20].

Bile acid dysregulation has also been implicated in other diseases, though the role of BSHs in their etiology is not well understood. A mouse model of nonalcoholic fatty liver disease (NAFLD) demonstrated that symptoms could be relieved by inhibiting bile acid reabsorption, leading to a reduction in conjugated bile acids, and a reduction in FXR antagonism [37]. BSH activity could potentially replicate this effect on the bile acid pool to improve clinical outcomes of NAFLD. Recent work has shown that liver tumorigenesis can be suppressed via an immune pathway that is stimulated by gut-derived primary, but not secondary, bile acids [38]. Modulating BSH activity to limit secondary bile acid metabolism could be a potential therapeutic for hepatic cancers. BSH activity has even been implicated in the development of autism spectrum disorder (ASD) [39]. Normal bile acid deconjugation and metabolism was able to promote healthy levels of inflammation and intestinal barrier function in a mouse model of ASD, possibly through FXR signaling, whereas shifts in the bile acid pool resulted in less deconjugation and less social mice with impaired cognition [39]. BSH activity also appears to be directly involved in the homeostasis of the GIT, specifically controlling circadian rhythms [20, 34, 40]. Given that a single bacterial enzyme can have a profound impact on human health and disease, there are many unanswered questions regarding BSH biology that need to be addressed before this enzyme can be used as a therapeutic tool.

### Future directions and outstanding questions

1. Can BSHs be used to alter the bile acid pool to rationally alter the gut microbiota and shape host physiology?
2. Are BSHs required for general bacterial fitness and competition for nutrients? Is this context dependent? What happens in the face of inflammation and other disease states?
3. Can BSHs be engineered with increased activity and substrate specificity for certain bile acids? What amino acid residues are important for determining bile acid substrate specificity?
4. How do BSHs with different activity and substrate specificity alter the bile acid pool, microbiota, and host response in different regions of the GIT? How will BSHs be delivered to the different regions of the GIT?
